# Service availability and readiness to provide comprehensive emergency obstetric and newborn care services in post-conflict at North Wollo Zone hospitals, Northeast Ethiopia: mixed survey

**DOI:** 10.1186/s12913-023-09165-5

**Published:** 2023-03-01

**Authors:** Misganaw Guadie Tiruneh, Eneyew Talie Fenta, Amare Mebrat Delie, Seteamlak Adane Masresha, Semira Muhidin Mustofa, Atitegeb Abera Kidie, Tsion Kokeb Kodo, Tadele Fentabil Anagaw

**Affiliations:** 1 School of Public Health, College of Health Sciences, Woldia University, Woldia, Ethiopia; 2 Department of Public Health, College of Medicine and Health Sciences, Injibara University, Injibara, Ethiopia; 3 Department of Health Promotion and Behavioural Science, School of Public Health, College of Medicine and Health Sciences, Bahir Dar University, Bahir Dar, Ethiopia

**Keywords:** Comprehensive emergency obstetric and newborn care services, North wollo zone, Public hospitals, Service availability and readiness

## Abstract

**Introduction:**

Countries with humanitarian crises and fragile conditions contribute to 61% of the global burden of maternal mortality. Emergency Obstetric and Newborn Care (EmONC) services reduce direct obstetric complications, which cause approximately 70–80% of maternal deaths and 10% to 15% of neonatal deaths. Therefore, this study was aimed to assess the service availability and readiness to provide comprehensive emergency obstetric and newborn care services in post-conflict at North Wollo Zone hospitals, Northeast Ethiopia.

**Methods:**

A facility-based mixed cross-sectional study design was conducted from May 10 to May 25, 2022, among North Wollo zone hospitals. Quantitative data were collected by using structured interviewer-administered questionnaires with observation and record review, entered by using Epi Data Version 4.6, and exported to SPSS 25 for analysis. Qualitative data were collected by key informant interviews and analyzed through thematic analysis. A descriptive data analysis was done to analyze the study variables.

**Results:**

Only three of the six hospitals (Woldia, Shediho Meket, and Saint Lalibella) performed all signal functions of comprehensive emergency obstetric and newborn care in the preceding three months. Cesarean section was the least performed signal function in post-conflict. The overall readiness to provide comprehensive emergency obstetric and newborn care services was 77.7%. Only one of the six hospitals had sufficient blood without interruption, and three of the six facilities had done screening for hepatitis B, HIV, and syphilis. Lack of supplies, equipment, and drugs were the challenges for the performance of EmONC signal functions.

**Conclusions:**

Post-conflict availability and readiness for comprehensive emergency obstetric and newborn care services in the North Wollo Zone was suboptimal. Shortage of medical supplies, equipment and emergency transportation was the challenges to provide these services. Thus, the hospital decision makers should strengthen leadership commitment, which focuses on recovering and rebuilding the destructed hospitals with resource mobilization and support.

## Introduction

Conflict has been described as a public health problem due to its negative impact on health systems and population health [1]. Conflict has a significant impact on maternal and child health service utilization both during and after the conflict [2–5]. Besides, it causes interruption of maternal and prenatal care services, which leads to the late detection of complications such as preeclampsia and eclampsia [5].

Since from November 2020, Ethiopia has suffered an armed conflict between Tigray People Liberation Front (TPLF) armed forces and the federal government in the northern part of the country [6]. Although the federal government declared a unilateral ceasefire in June 2021, the conflict expanded to the Amhara and Afar regions and caused destruction of the health facilities in the affected areas [7]. The armed conflict makes women much more vulnerable and exposes them to different health problems by substantially reducing the availability and accessibility of maternal care through curfews, closures, and blockades [2]. The number of maternal deaths in conflict affected states and fragile conditions contributes to 61% of the global burden of maternal mortality [8]. In 2020, the global maternal mortality ratio was 152 deaths per 100,000 live births and 302 deaths per 100,000 live births in Sub-Saharan Africa [9]. In Ethiopia, according to the 2017 Federal Ministry of Health report, 401 maternal deaths for every 100,000 live births and 33 neonatal deaths per 1000 live births occurred [10]. The provision of emergency obstetric and newborn care (EmONC) services reduces and treats direct obstetric complications that make up approximately 70–80% of maternal deaths and 10% to 15% of neonatal deaths [11].

Comprehensive emergency obstetric and newborn care (CEmONC) services are a set of nine vital life-saving interventions provided at hospitals when a woman or her newborn experiences serious complications [11]. Utilization of maternal and child health care services is among the priorities to reduce maternal mortality rates as part of the Sustainable Development Goals (SDG) objectives [12]. Ensuring availability and facility readiness is an important first step to improving utilization and quality of care [13]. However, poor availability of clinical services due to a lack of infrastructure, emergency transportation, and capacity in medical supplies has been identified as the challenges to provide essential emergency obstetric services in Ethiopia [14]. Unfortunately, the disruptive effect of conflict exacerbates health personnel shortages, damages health infrastructure, and weak supply chains, all of which contribute to increased vulnerability to adverse obstetric complications and mortality [15]. Thus, it is crucial to ensure that facilities are sufficiently resourced and equipped to provide essential maternal and newborn care [14, 16].

In Ethiopia, there are several studies which assessed the availability of Emergency Obstetric and Newborn care (EmONC) Services before the occurrence of the Northern Ethiopia conflict [14, 17–21]. The Ethiopian EmONC assessment report revealed that, even though 921 EmONC facilities were recommended, only 370 facilities were fully functioning as EmONC and only 148 facilities were fully CEmONC, compared to the recommended 184 CEmONC facilities in Ethiopia [22]. Furthermore, the report revealed that only 101 of the recommended 208 facilities were EmONC functional in Amhara region [22].

In Dire Dawa, out 6 facilities expected to offer CEmONC service only one was qualified as CEmONC health facility [17]. A study in the Oromia region also showed that there were only 0.24 CEmONC facilities per 500,000 people [19]. However, there is sub-optimal knowledge on the availability and readiness to provide CEmONC services in post-conflict at North Wollo Zone hospitals. Therefore, this study was aimed to assess the service availability and readiness of hospitals to provide CEmONC services in post-conflict at North Wollo zone. The evidence from this study will contribute to the recovery of fragmented health facilities as part of the post-conflict effort to improve maternal and neonatal health in the North Wollo zone.

## Methods and materials

### Study design and setting

A mixed-method facility assessment was conducted from May 10 to May 25, 2022, in North Wollo Zone hospitals, 5 months after the cessation of the conflict. North Wollo is located in the Amhara region of Ethiopia with Woldia as the central town. Woldia is found at 521 km North away from Addis Ababa and 360 km East from Bahirdar. There are six governmental hospitals in North Wollo zone which are found in the capitals of their respective districts. These are Woldia comprehensive specialized Hospital, Kobo Hospital, Lalibela Hospital, Shediho Meket Hospital, Wadila Hospital, and Mersa Hospital.

### Study population

All hospitals found in North Wollo Zone were included in this study.

### Sample size and sampling procedure

The emergency obstetric care monitoring handbook [11] recommends that if there are 25 or fewer hospitals, study all of them, and if there are more than 25 hospitals select a sub-set as many as possible that should represent at least 30%. Therefore, based on the recommendation of emergency obstetric care monitoring handbook, all of the six hospitals in the study area were included.

Purposive sampling was used to select the hospital managers and the head of midwives as key informants for the qualitative part of the study since they are supposed to be experienced and rich in information related to the CEmONC service availability and readiness until saturation of ideas.

### Measurement of variables

CEmONC service availability: is the physical presence of the services related to the provision of CEmONC [23]. It was measured based on whether the following nine signal functions have ever been carried out within the facility at least once during the past 3 months: ‘parenteral administration of antibiotics’, ‘parenteral administration of oxytocics’, ‘parenteral administration of anticonvulsants’, ‘assisted vaginal delivery’, ‘manual removal of placenta’, ‘manual removal of retained products of conception’, ‘neonatal resuscitation’, ‘caesarean sections ‘and ‘blood transfusions’ [4, 11].

CEmONC service readiness: refers to the ability of hospitals to offer CEmONC services, and the capacity to provide the service measured through consideration of 20 tracer items of four domains that include trained staff and guidelines, equipment, diagnostic capacity, and medicines and commodities [23].

The first domain was staff and training, which was measured by four tracer items (the presence of guidelines for CEmONC, the presence of at least one CEmONC staff trained within the past two years, a health worker who can perform caesarean section present in the facility or on-call 24 h a day, and the presence of anesthetist in the facility or on-call 24 h a day). The second domain was equipment which had five tracer items (anesthesia equipment, resuscitation table, incubator, oxygen with functional flow meter and key connecting tubes available at all times during the 3 past months and spinal needle). The third domain diagnostic capacity was measured by two indicators (blood typing and cross-match testing) and the fourth domain medicines and commodities had nine indicators; it contains (sufficient blood supply without interruption in last three months, blood supply safety, 5% lidocaine, injectable epinephrine, inhalational halothane, injectable atropine, thiopental powder, suxamethonium bromide, and ketamine injection) [23].

CEmONC service readiness score: was computed as the mean availability of service specific tracer items in four domains [23]. The domain score was calculated as dividing the total number of items available by the total number of items in the domain. Finally the CEmONC service readiness score was computed by dividing the sum of the domain score to the number of domains [23].

### Data collection procedure

The quantitative data were collected by using the paper based WHO health facility service availability and readiness (SARA) assessment tool [23]. The tool was contained service availability and service specific readiness questions with Yes / No responses. The responses for CEmONC service readiness questions were verified by direct observation, and data for CEmONC service availability (the presence of nine signal functions) were collected from the maternal delivery register by document review. The quantitative data were collected by three midwives and supervised by two public health professionals.

The qualitative data were collected by in-depth interviews with key informants. It was conducted by the principal investigator through a face-to-face interview with the hospital manager and the midwife head using unstructured, open-ended questions. The interview was conducted in Amharic by the local language, with the aid of audio recorder. The principal investigator took notes during the interview. In cases of ambiguity, the principal investigator explains the issues promptly. All interviews recorded were transcribed verbatim.

### Data quality assurance

One day of training was given for data collectors and supervisors about the objective of the study and data collection technique. The supervisors were check the completeness and consistency of the collected data. The investigators were checking the completeness and consistency of all questionnaires daily.

### Data management and analysis

Data were entered, cleaned, and coded using Epi Data Version 4.6 and analyzed using Statistical Package for Social Sciences (SPSS) Version 25. Descriptive data analysis was done to describe the variables under the study. Different frequency tables, graphs, and descriptive summaries were used to describe the study variables.

The principal investigator transcribed the qualitative data verbatim from the audio recording and translated it into English. The transcription was rechecked with the original data to confirm consistency by investigators. The data were read and re-read by investigators. Open code version 4.02 qualitative data analysis software was used for data coding and analysis. Codes were identified and grouped into themes inductively.

## Result

### CEmONC service availability in hospitals

Among the surveyed six facilities, it was found that 3 (50%) health facilities (Woldia, Shediho Meket, and Saint Lalibela) performed all CEmONC signal functions in the past three months of post-conflict. Cesarean section was the least performed signal function (3/6), followed by blood transfusion (4/6) and assisted vaginal delivery (5/6) (Table 1).

**Table 1:** Performance of signal functions in the past 3 months of post-conflict in North Wollo zone hospitals, Northeast Ethiopia, 2022

S. No	Signal functions	Frequency (%)
1	Parenteral administration of antibiotics	6 (100%)
2	Parenteral administration of oxytocic drugs	6 (100%)
3	Parenteral administration of anticonvulsants	6 (100%)
4	Assisted vaginal delivery	5 (83.3%)
5	Manual removal of placenta	6 (100%)
6	Manual removal of retained products	6 (100%)
7	Neonatal resuscitation	6 (100%)
8	Blood transfusions	4 (66.7%)
9	Cesarean sections	3 (50%)

### Availability of important supportive items for CEmONC services

#### Staff training & guidelines

All of the hospitals reported that they had health workers who could perform cesarean section and provide anesthetics. Also, four of the six (66.7%) of facilities reported having at least one staff member who had received refresher training in CEmONC services within the past 2 years (Table 2).

**Table 2:** Indicators of readiness to provide CEmONC services in post-conflict in North Wollo Zone public hospitals, Northeast Ethiopia, 2022

Indicators	n (%) of facilities
**Staffs & Guidelines**	
Guidelines for CEmONC	5 (83.3%)
Availability of CEmONC trained staff	4 (66.67%)
Availability of staff for cesarean section	6 (100%)
Presence of anesthetist	6 (100%)
**Equipment**	
Anesthesia equipment	4 (66.67%)
Resuscitation table	5 (83.3%)
Incubator	4 (66.67%)
Oxygen with functional flow meter and key connecting tubes available at all times during the 3 past months	5 (83.3%)
Spinal needle	3 (50%)
**Diagnostics**	
Blood typing	6 (100%)
Cross match testing and centrifuge	4 (66.67%)
**Medicines and Commodities**	
Blood sufficiency	1 (16.7%)
Blood screened for HIV, Syphilis, Hepatitis B, and Hepatitis C	3 (50%)
Lidocaine 5%	6 (100%)
Epinephrine (injectable)	5 (83.3%)
Halothane (inhalation)	5 (83.3%)
Atropine (injectable)	6 (100%)
Thiopental (powder)	4 (66.67%)
Suxamethonium bromide (powder)	3 (50%)
Ketamine (injectable)	5 (83.3%)

*CEmONC* comprehensive emergency obstetric and newborn care

#### Equipment and diagnostics

All of the six hospitals can perform blood typing to identify the blood group. The majority of the facilities (5/6) were equipped with resuscitation table and functional oxygen with flow meter. But, only half (50%) of the facilities were equipped with spinal needle (Table 2).

#### Medicines and commodities

Regarding the availability of essential medicines, all of the facilities had 5% lidocaine. Similarly, more than 80% of facilities had injectable epinephrine, injectable ketamine, and inhalational halothane. However, only one of the six hospitals had blood without interruption in the last three months (Table 2).

#### Readiness to provide CEmONC services

**Fig. 1:**
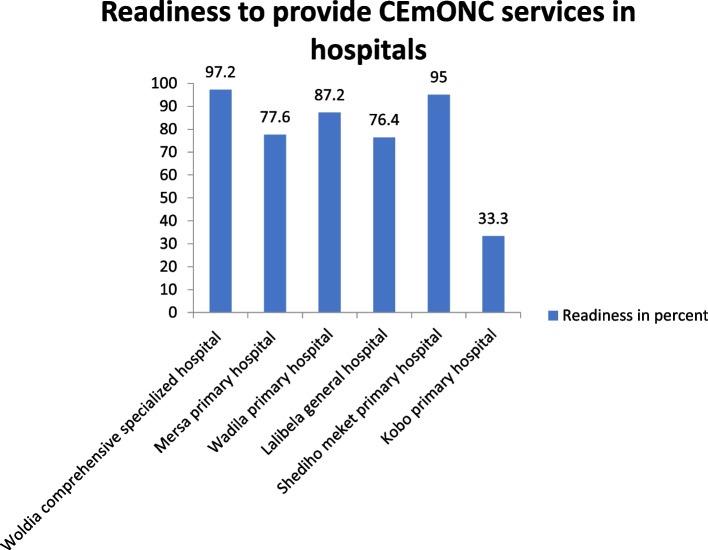
Percentage of hospitals readiness score to provide CEmONC services in post-conflict in North Wollo Zone public hospitals, Northeast Ethiopia, 2022

The mean availability of 20 tracer items for CEmONC services in post-conflict was 77.7%. Woldia comprehensive specialized hospital had the highest (97.24%) CEmONC services readiness score, whereas Kobo primary hospital had the lowest total CEmONC service readiness score of 33.3% (Fig. 1).

## Qualitative findings

### Socio-demographic characteristics of the participants

About 11 key informants (5 hospital managers and 6 MCH unit heads) were participated in the in-depth interview of key informants. All of the participants were male and aged between 32 to 37 years old. More than 90% of participants had experience of more than five year. The interviews were held using a semi-structured interview guide with probing questions related to CEmONC services.

Three central themes were identified: availability of CEmONC services in post-conflict, challenges for provision of CEmONC services in post-conflict and impact of conflict on CEmONC services.

### Theme 1: Availability of CEmONC services in post-conflict

Majority of the respondents mentioned that most of the basic emergency obstetric care services signal functions were reopened and resumed immediately after cessation of the conflict. The participants also reported that they started the obstetric services with oxytocin immediately after the area was liberated from the armed forces. Even though, the services were started by the basic supplies [oxytocin, some parenteral antibiotics and parenteral anticonvulsants], the signal functions were not fully available. Half of the participants noted that cesarean section had not started yet.

A 37-years old male Maternal and Child Health (MCH) unit head said that *“When we had just entered to the hospital, equipment was completely destroyed, and they took what they had taken. As soon as we entered, we went into cleaning and burning of all the destroyed papers. We started packing and repairing the items. The first thing we needed was oxytocin to care & deliver mothers’ during an emergency”.*

A 34-years old male MCH head in one of the district hospitals said that *“More or less, the EmONC services were given but the services were not fully functional. Since all medical equipment and supplies were destroyed by the conflict the operation room has not started yet”.*

A 35-years old male manager of the hospital noted that *“After the area was free and controlled over by government forces, the first activity was an assessment of how much medical equipment was destroyed? How much was stolen? We did a comprehensive assessment of the facility with experts from the regional health bureau to find out what was needed for recovery, after which we decided services we could start initially. In that sense, it was obvious that the emergency maternal and child health services should be started early, because maternity care by itself is an emergency service”.*

### Theme 2: Challenges for provision of CEmONC services in post-conflict

#### Human resource

The majority of the respondents stated that there was no shortage of health care providers in maternity units for obstetric care. The participants also reported that transfers to each other as well as transfers by the regional health bureau were cancelled from the beginning.

A 33-years old male MCH unit head said that *“From the beginning, we first returned to work, transfer to each other as well as transfer by regional health bureau was cancelled and all professionals kept in place. Because, allowing transfer with the current condition creates.*

additional problem. Even it is difficult to adapt to the situation when you are rotating from ward to ward. Everyone is on job without getting leave period”.

Additionally, almost all of the respondents mentioned that the armed conflict makes the health professionals committed for work and use their maximum effort for their community.

“The employees were more motivated than before, even with the excessive number of cases from the neighboring districts” (a 32-years old male MCH unit head).

In contrary, one third of the participants mentioned that their facilities were overloaded by the referral cases from the surrounding health facilities due to sever destruction of the facilities and needs for operation. Accordingly, the number of maternal cases were higher than the professionals in the maternal unit.

A 32-years old male MCH unit head said that *“After the conflict, the neighboring district health facilities were not fully functional. Due to this, we were loaded with excessive case flow; we think they [mothers] might be unsatisfied as their expectation”.*

One of the respondent in one of the district hospital also mentioned that *“…The other issue, the healthcare providers are not motivated & initiated as before. Therefore, the health professionals need psychosocial support and motivation…. I'm still not happy. I'm scared”.* (a 34-years old male MCH unit head).

#### Infrastructure

All of the respondents reported that all of the health facilities were damaged during the armed conflict. Most the respondents also stated that the electric power was the main problem to perform the nine signal functions.

In support of this a 35 years old male manager in one of the district hospitals expressed the challenge to provide the CEmONC signal functions *“…but the main problem strangled us is power shortage. There are drugs expired due to lack of power. For example, we cannot bring blood because of lack of power, so we cannot perform the operation as needed”.*

A 37-years old male MCH unit head also said that *“In my opinion, the main obstacle for maternal and obstetric service is the electric power. ….”.*

#### Ambulance

All of the participants reported that all of the hospitals had shortage of ambulance and majority of the ambulances were looted. The participants also reported that most ambulances are under maintenance which was damaged during the conflict. According to the participants this situation leads the life of mothers at risk. The participants also noted that where mothers referred to the next higher health facility they have to make their own way to take contract vehicles.

A 35-years old male manager said that *“We have no ambulance totally due to this mothers are at risk of dying. Even if we tried to refer, the risk is much higher”.*

A 37-years old male MCH unit head also stated that *“….For example, if we have a mother who needs operation, we have to refer her. Especially if multiple mothers come to us, even if we send the first mother by command post vehicles, we have a risk to loss the second one. There are cases that we do by taking risks…”.*

#### Medical supplies and drugs

Most of the interviewees noted that shortage of medical supplies and equipment were the great challenge to the provision of CEmONC services. Specially, about one third of participants were noted medical equipment used for cesarean section like anesthesia machine, operation table, ultrasound and other laboratory supplies are not available.

A 34-years old male manager in one of the district hospitals said that *“There is no emergency CS because the operation room is not started yet due to lack of operation and anesthesia machines which caused us a lot of problems”.*

A 32-years old MCH unit head in one of the district hospitals also said that *“The problem is that the operation has not started yet, we are treating others well. Mothers are just being suffering due to refer for emergency cesarean section while it can be done here”.*

Shortage of incubator and radiant warmer were also reported by half of the participants. In addition, most of the participants also mentioned that advanced laboratory equipment like chemistry machine, CBC machine and hormonal analysis tests were looted and not replaced yet.

Besides, half of the participants also reported that shortage of basic laboratory investigations like Hgb, syphilis and hepatitis tests also challenges for the provision of CEmONC services.

A 32-years old MCH unit head noted that *“ In the laboratory service, hepatitis and syphilis tests are not being done since for the last one month. There is also a shortage of HIV test kits. A mother with a stat pack reactive will not get a full screening services”.*

Shortage of blood was also mentioned as one of the most important factor to provide the CEmONC services by majority of the respondents. Additionally absence of electric power and refrigerator were the obstacles to store blood for CEmONC service provision.

A 35-years old male manager from one of the district hospitals said that *“we cannot bring blood because of the lack of power, so we cannot perform operations for mothers as needed. If a mother needs blood we refer her”.*

Majority of the respondents reported that anesthesia drugs were not available in the facilities. Whereas about one third of the respondents mentioned that consumable basic antibiotics like ceftriaxone and gentamycin were also be the challenges for the provision of CEmONC services.

*“We lack anesthesia drugs for cesarean section in particular. We have also shortage of basic drugs like antibiotics because they are frequently consumables in related to conflict”*. (a 35-years old male manager).

### Theme 3: Impact of the conflict on CEmONC services

About one third of respondents noted that since there was a collapse of health services, mothers didn’t get regular antenatal care (ANC) follow up which in turn leads to bad obstetrics outcomes during delivery. Additionally, they mentioned that mothers developed psychological problems when they heared of noises and weapon sounds.

A 33-years old male MCH head in one of the hospitals said that “*A mother with congenital anomalies don’t know her case until she gave birth because she has never had an ultrasound check and has no ANC follow-up as well as TT vaccination. Therefore, lack of antenatal care follow up has a negative impact on our delivery outcomes. This has a profound effect on the psychology of community & family which means a pregnant mother who gave birth to a child with malformation and deformity results in serious psychological and social problem”.*

Increased the need of abortion among women who were raped during the conflict was also explained by one of the MCH unit head (a 37-years old male midwife); *“There are still many women who were raped and now want to access an abortion services. We are advising them not to have an abortion and to continue their pregnancy as much as possible as they are reaching to term”.*

The war causes difficulty in provision of free maternal services. Medicines and drugs were looted and destroyed.

A 35-years old male manager noted that *“maternal and child health services are free although the guideline states that free services will be reimbursed by the regional council but not implemented. It was only covered by the hospital and the hospital has been selling medicines to other patients and making money. It was not difficult to provide free maternal services before. But now the medicine has been stolen, the immovable has been destroyed from here. So now it is very difficult to provide free services. Because the amount of money you collect on credit is very small”.*

## Discussion

In this study, we assessed the availability and readiness of comprehensive EmONC services in post-conflict. The availability of nine signal functions for CEmONC is crucial to reduce maternal and neonatal mortality [24, 25]. This study showed that 50% of health facilities (Woldia comprehensive specialized hospital, Shediho Meket primary hospital, and Saint Lalibela general hospital) performed all CEmONC signal functions in the preceding three months.

This study finding was higher than a study conducted in Congo only 30% of health facilities were fully functional as CEmONC [4]. The possible explanation for this difference might be due to the small sample size of this study or the difference in study setting. In the previous study the facilities were selected purposively in three zones with the ‘red flag’ proximity (the deadliest) conflict which may decreases the performance of CEmONC signal functions [4].

However, the finding of this study was lower than a study done in three humanitarian settings (Burkina Faso, Democratic Republic Congo (DRC) and South Sudan), which found that, except one facility in Burkina Faso, all hospitals provided all signal functions of CEmONC [26]. The possible explanation for the difference might be due to the humanitarian aid difference for the obstetric care services, in which the previous study was conducted to know the progress of reproductive health services through humanitarian aids. This implies that the provision of humanitarian aid is crucial for the performance of CEmONC services in post-conflict situations.

This study finding was also lower than the 2016 Ethiopian EmONC final report in the Amhara region, which reported that 58% of facilities were functioning as CEmONC [22]. The possible explanation for the difference might be due to the study period which was done in post-conflict, the equipment and medical supplies to perform the CEmONC signal functions might have been possibly destroyed. This implies that providing medical supplies and equipment is needed for the fast recovery of destroyed hospitals to perform the emergency obstetric and newborn care services.

In this study, cesarean section was performed in 50% of hospitals, which was lower than the national Ethiopian service availability and readiness report of 86% [21] and study finding in Ethiopian developing regions [27]. The difference might be due to the study period. This study also showed that 66% of hospitals performed blood transfusions which was comparable with the study finding in Ethiopian developing regions, 66.6% [27]. Whereas the finding of this study was lower than the national Ethiopian service availability and readiness report 82% [21].

In this study, 50% of the surveyed facilities performed all nine signal functions in the past three months of post-conflict, with cesarean section and blood transfusion being the least frequently performed. Inadequate availability of these important major components of CEmONC might lead to unnecessary delay to prevent or intervene prolonged labor, which is one of the leading causes of maternal mortality [28]. However, all health facilities had carried out at least one of the nine signal functions during the last three months of post-conflict before the survey.

In this study, cesarean section was the least performed signal function, which was contradictory to the study conducted in the DRC [4] and the Ethiopian emergency obstetric care services report, which found that assisted vaginal delivery was the least performed signal function [22]. The difference might be due to the study population in which these studies include both EmONC services and more basic emergency obstetric and newborn care facilities, where assisted vaginal delivery needs skilled personnel and advanced equipment from the seven basic EmONC signal functions. Moreover, in this study, the conflict might cause destruction of cesarean section equipment and medical supplies, which in turn would decrease the performance of cesarean section.

This study also found that basic emergency obstetric and newborn care service signal functions, except assisted vaginal delivery, were most frequently performed in the post-conflict. The qualitative interview also supported that the facilities start obstetric care with the essential oxytocin drug. The possible explanation might be that relatively the basic emergency obstetric care may not need the advanced medical equipment for performance.

The qualitative finding revealed that lack of equipment, drugs, and supplies were the main reasons for the non-performance of all signal functions. In agreement with this result, a study conducted in Nigeria and DRC reported that lack of medical supplies, equipment, and drugs were the barriers to EmONC service provision [4, 29]. The possible explanation might be due to the study period, which was done in post-conflict, the equipment to perform the signal functions might have been destroyed.

Facility readiness is an important aspect that shows a facility’s commitment to ensuring the cumulative availability of components required to provide a specific service [25]. This study showed that the mean readiness to provide CEmONC services was 77.7%. This study finding was higher than the Ethiopian SARA report of Amhara region, 69% [21]. The possible explanation for the difference might be that due to the donors focus for recovery from governmental and non-governmental organizations, or it might be due to the small size of the study.

This study reported that Woldia comprehensive specialized hospital had the highest (97.24%) CEmONC services readiness score, and Kobo primary hospital had the lowest average CEmONC service readiness score of 33.3%. The possible explanation might be due to the long duration of the conflict in Kobo compared to Woldia.

This study also reported that only one of the six (16%) facilities had sufficient blood and three (50%) of facilities screened blood for HIV, syphilis, and hepatitis, which was lower than the 2016 Ethiopian SARA report of Amhara region, which reported that 20% and 94% respectively [21]. The possible explanation might be due to the study period since this study conducted in post-conflict which may causes destruction of medical supplies and equipment.

The qualitative finding stated that the need for abortion was high after the cessation of the conflict. The possible explanation might be the due to high incidence of sexual violence during the conflict [30, 31].

The qualitative interview also found that there is no shortage of human resources; professionals were available at all times in all hospitals for cesarean section and anesthesia, and the conflict also makes health professionals committed to work.

Furthermore, the qualitative interview findings revealed that the challenges to providing CEmONC services in post-conflict included a lack of medical supplies and equipment, drugs, and emergency transportation. In facilities with lowest readiness might be due to looted and destroyed medical equipment & supplies in these facilities that may contribute to insufficiencies and low quality of obstetric care in health facilities. Thus, the provision of medical supplies and drugs for hospitals is vital for the readiness of hospitals to provide the lifesaving comprehensive emergency obstetric and newborn care services consistently.

## Limitation of the study

This study didn’t include the health centers which provide the basic emergency obstetric and newborn care services, nor did it the client’s response about the services. The data for CEmONC service availability from registers might be incomplete and we only use the service specific tracer items for the assessment of CEmONC service readiness which doesn’t include all the necessary supplies for obstetric care. The study also didn’t measure the knowledge and capacities of care providers.

## Conclusion

The post-conflict availability and readiness to provide the CEmONC services was suboptimal. Lack of medical supplies and equipment, drugs and shortage of emergency transportation were the challenges to provide the comprehensive emergency obstetric and newborn care services in post-conflict. Although mothers have the desire and decision to seek the services, these challenges affect maternal health through delay in transportation and receiving care at the health facility.

We recommend the hospital decision makers to cultivate strong political and leadership commitment which focuses on recovering and rebuilding of the destructed hospitals with resource mobilization and support. Amhara regional health bureau should strengthen the provision of medical supplies and drugs for CEmONC services. Further studies including the basic emergency obstetric and newborn care services with client interview should also be considered by other researches.

## Data Availability

All the data supporting the findings are within the manuscript. Additional detailed information and raw data are available from the corresponding author with reasonable request.

## Abbreviations

ANC: Antenatal care
BEmONC: Basic Emergency Obstetric and newborn care
CEmONC: Comprehensive emergency obstetric and newborn care
EmONC: Emergency obstetric and newborn care
MCH: Maternal and Child Health
SARA: Service availability and readiness (SARA) assessment
SDG: Sustainable Development Goal
